# HLA-DR Alpha 2 Mediates Negative Signalling via Binding to Tirc7 Leading to Anti-Inflammatory and Apoptotic Effects in Lymphocytes In Vitro and In Vivo

**DOI:** 10.1371/journal.pone.0001576

**Published:** 2008-02-13

**Authors:** Grit-Carsta Bulwin, Stephanie Wälter, Mirko Schlawinsky, Thomas Heinemann, Anke Schulze, Wolfgang Höhne, Gerd Krause, Wiltrud Kalka-Moll, Patricia Fraser, Hans-Dieter Volk, Jürgen Löhler, Edgar L. Milford, Nalân Utku

**Affiliations:** 1 CellAct Pharma GmbH, Berlin, Germany; 2 Kekulé-Institut für Organische Chemie und Biochemie, Universität Bonn, Bonn, Germany; 3 Institut für Biochemie, Charité Universitätsmedizin Berlin, Campus Mitte, Berlin, Germany; 4 Leibniz-Institut für Molekulare Pharmakologie, Berlin, Germany; 5 Medizinische Mikrobiologie, Klinikum der Universität zu Köln, Köln, Germany; 6 Division of Rheumatology, Department of Medicine, Brigham and Women's Hospital, Boston, Massachusetts, United States of America; 7 Institut für Medizinische Immunologie, Charité, Campus Mitte, Humboldt-Universität zu Berlin, Berlin, Germany; 8 Molekulare Pathologie, Heinrich-Pette-Institute, Hamburg, Germany; 9 Renal Division, Department of Medicine, Brigham and Women's Hospital, Boston, Massachusetts, United States of America; New York University School of Medicine, United States of America

## Abstract

Classically, HLA-DR expressed on antigen presenting cells (APC) initiates lymphocyte activation via presentation of peptides to TCR bearing CD4+ T-Cells. Here we demonstrate that HLA-DR alpha 2 domain (sHLA-DRα2) also induces negative signals by engaging TIRC7 on lymphocytes. This interaction inhibits proliferation and induces apoptosis in CD4+ and CD8+ T-cells via activation of the intrinsic pathway. Proliferation inhibition is associated with SHP-1 recruitment by TIRC7, decreased phosphorylation of STAT4, TCR-ζ chain & ZAP70, and inhibition of IFN-γ and FasL expression. HLA-DRα2 and TIRC7 co-localize at the APC-T cell interaction site. Triggering HLA-DR - TIRC7 pathway demonstrates that sHLA-DRα2 treatment inhibits proinflammatory-inflammatory cytokine expression in APC & T cells after lipopolysaccaride (LPS) stimulation in vitro and induces apoptosis in vivo. These results suggest a novel antiproliferative role for HLA-DR mediated via TIRC7, revise the notion of an exclusive stimulatory interaction of HLA-DR with CD4+ T cells and highlights a novel physiologically relevant regulatory pathway.

## Introduction

TIRC7 (TCIRG1) is a seven transmembrane protein induced early after allo-activation of human lymphocytes [1], [2]. Targeting of TIRC7 with antibodies decreased IL-2 transcription and inhibited the release of IFN-γ, but not IL-10 in vitro and in vivo [1]–[3]. Cell surface expression profiles and results obtained from antibody blocking studies suggested that TIRC7 interacts with a ligand at the cell surface. Here we report that the HLA-DR alpha 2 domain is a ligand for TIRC7. HLA-DR molecule consists of an alpha and beta chain expressed on antigen presenting cells and activated T cells. Binding of HLA-DR to T cell receptor on CD4+ T cells is known to initiate immune activation and accordingly HLA-DR molecules are considered to be immune stimulatory.

We extend this view by providing evidence for an important novel negative regulatory role of HLA-DR executed via binding with its non-polymorphic alpha 2 domain to TIRC7, a negative regulator of immune activation expressed on activated lymphocytes. Binding of HLA-DR alpha 2 to TIRC7 delivers antiproliferative signals to lymphocytes which is not solely restricted to CD4+ cells. Induction of the HLA-DR alpha 2 - TIRC7 pathway leads to activation of the intrinsic apoptotic pathway resulting in apoptosis of CD4+ and CD8+ lymphocytes. The downregulatory mechanism of the immune response is associated with SHP-1 recruitment and include the inhibition IFN-γ expression, phosphorylation of STAT4, TCR-ζ chain, ZAP70 and expression of FasL in T cells. Ligation of TIRC7 using soluble HLA-DRα2 (sHLA-DRα2) causes apoptosis in lymphocytes via activation of caspase 9 and 7. TIRC7 and HLA-DR are co-localized at the site of T cell - APC interaction. Accordingly, targeting of TIRC7 with sHLA-DRα2 controls proinflammatory cytokine release in APC and T cells in vitro. Physiological relevance of TIRC7 and HLA-DR α2 binding is shown in acute inflammatory setting in vivo after LPS. Treatment of mice with sHLA-DRα2 inhibits APC and T cell cytokine expression and induces apoptosis in vivo underlining the physiological importance of TIRC7-HLA-DR alpha 2 binding. Notably, the modulation of the HLA-DR alpha 2 - TIRC7 pathway in vivo allows to reduce significantly the inflammatory response and cytokine release induced by APC-T cell interaction during immune activation. Thus, our results demonstrate that binding of HLA-DR alpha 2 to TIRC7 at the APC-T cell interaction side provides negative signalling events during immune activation leading to downregulation of the immune response.

## Results

### TIRC7 protein binds to the alpha 2 domain of HLA-DR molecule

**Figure 1:**
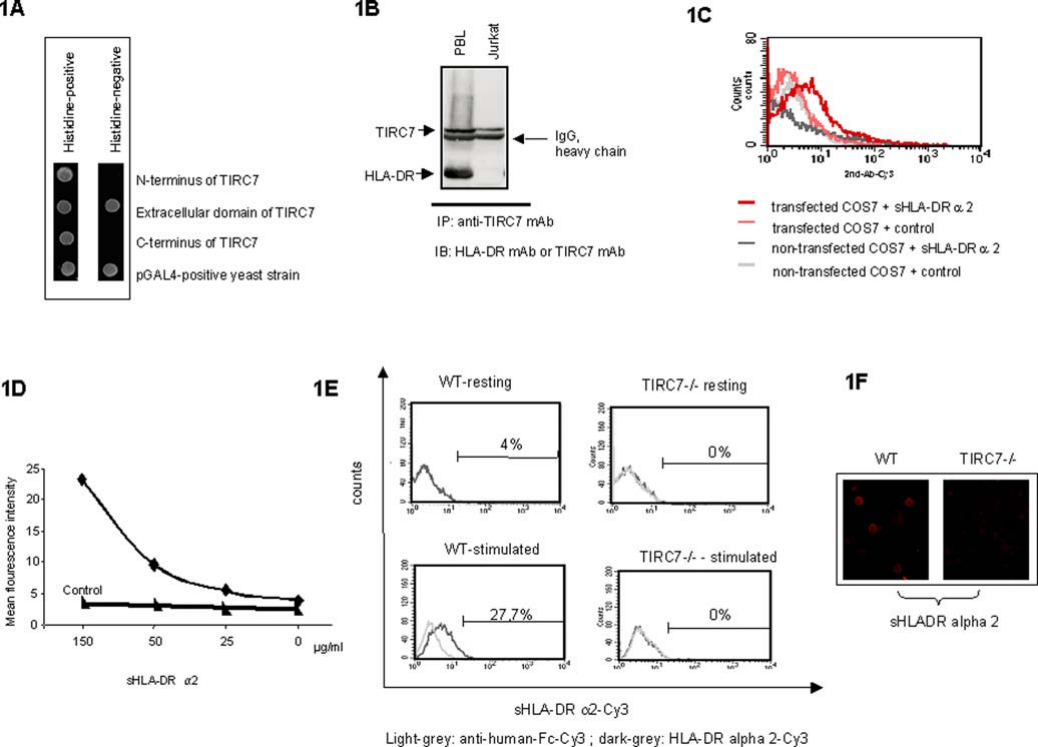
HLA-DR alpha 2 domain interacts with TIRC7 protein. A. Strain AH109 carrying GAL4 activation domain, fused to cDNA containing HLA-DR, was tested for interaction with indicated domains of TIRC7 (N-terminus aa 1-173, large extracellular domain aa 438-512 or C-terminus aa 586-614) as bait. The growth of combined clones on Histidine-negative agar plates indicate a specific interaction between the HLA-DR alpha 2 and TIRC7 extracellular domain whereas no interaction between HLA-DR alpha 2 to the C – terminal or N- terminal domain of TIRC7 was observed. The growth of colonies on Histidine positive agar plates which represents a positive control, is shown in the left panel. B. Lysates were prepared from 1 h allo-activated PBL and Jurkat cells. Lysates were immunoprecipitated (IP) with anti-TIRC7 mAb and immunoblotted (IB) with specific antibody against HLA-DR protein or TIRC7 in denaturing conditions. Co-precipitation of TIRC7 and HLA-DR is observed in PBL whereas only TIRC7 was precipitated in HLA-DR negative Jurkat cells. C. COS7 cells were transiently transfected with a TIRC7-*myc* fusion protein vector construct, incubated with sHLA-DRα2 and stained with secondary anti-human Fc protein-Cy3 conjugated mAb. Flow cytometry analysis revealed that sHLA-DRα2 solely binds to TIRC7 transfected COS7 cells and fail to bind to non-transfected COS7 control cells. Shown is one experiment out of four. D. COS7 cells transfected with TIRC7-*myc* fusion protein showed concentration-dependent binding of sHLA-DRα2 (0, 25, 50, 150 µg/ml) using direct immunofluorescence method. No binding of control protein was observed in transfected COS7 cells. Shown is one experiment out of three. E and F. TIRC7 deficient and wild-type mouse splenocytes were isolated and either stimulated with PHA for 48 h or remained unstimulated. Cells were incubated with either human HLA-DR alpha 2 or human control protein prior to flow cytometry (E) or confocal microscopic analysis (F) using anti-human Fc-specific Cy3 as secondary antibody. The results show that HLA-DR alpha 2 solely binds to stimulated WT cells in flow cytometric and microscopic analyses. Shown is one experiment out of three, respectively.

To investigate the ligand(s) interacting with TIRC7 we established a yeast two-hybrid screen using a cDNA library of allo-activated human peripheral blood lymphocytes (PBL). Constructs of the N-terminal, C-terminal sequence stretch, and large extracellular loop of TIRC7 were fused to a Gal4-binding domain and used as bait. While there were no interactions found with the N-terminal or C-terminal domains of TIRC7, a clone interacting with the large extracellular loop of TIRC7 was identified which contained the sequence of human HLA-DR alpha 2 (Figure 1A). This binding was confirmed by an anti-TIRC7 mAb co-precipitating HLA-DR alpha 2 from lysates obtained from allo-activated PBL, using specific anti-HLA-DR mAb for immunoblotting. In contrast, in lysates of the Jurkat cell line which is not expressing HLA-DR anti-TIRC7 mAb only TIRC7 was precipitated (Figure 1B). Similar results were obtained utilizing a polyclonal antibody recognizing TIRC7 [1] which also co-precipitated HLA-DR alpha chain as confirmed by protein sequencing (data not shown).

### Soluble HLA-DR alpha 2 specifically binds to TIRC7 and the interaction modulates IFN-γ expression and STAT4 signaling

The ability of HLA-DR alpha 2 to interact with TIRC7 was further analysed in binding studies using a soluble HLA-DR alpha 2 fusion protein consisting of the entire human HLA-DR alpha 2 domain linked to an IgG1 Fc protein and expressed in COS7 cells. The ability of sHLA-DRα2 to bind to TIRC7 expressed on the cell membrane was tested in COS7 cells which were transiently transfected with a TIRC7 cDNA fused to a *myc*-tagged expression vector at the extracellular carboxy terminus [1]. Flow cytometry using an anti-Fc protein antibody revealed that human sHLA-DRα2 exclusively bound to the surface of TIRC7-expressing COS7 cells whereas no binding was observed in non-transfected control COS7 cells (Figure 1C). The binding of sHLA-DRα2 to TIRC7 expressed in COS7 cells was shown to be concentration-dependent (Figure 1D). The binding specificity was confirmed when splenocytes of wild-type and TIRC7 deficient mice were stimulated with PHA and incubated in the presence and absence of sHLA-DRα2. Flow cytometry and microscopy revealed that sHLA-DRα2 binds solely to splenocytes from wild type mice whereas no binding was observed on splenocytes from TIRC7 deficient mice (Figure 1E and F).

**Figure 2:**
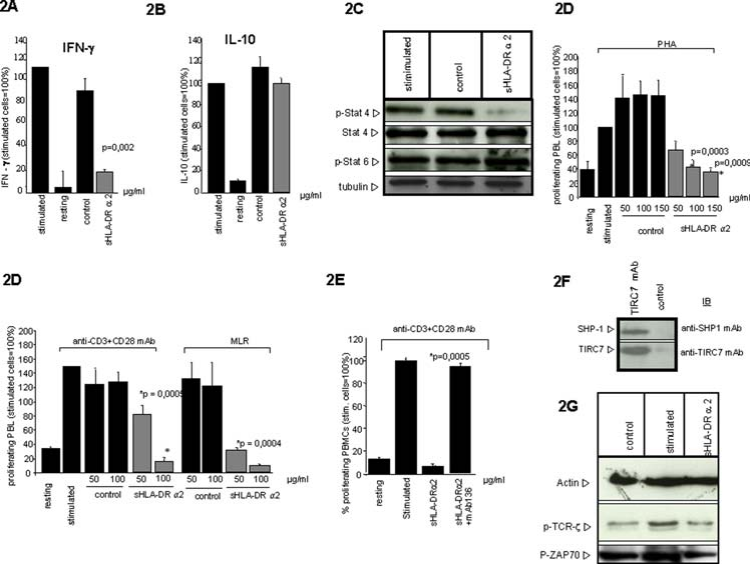
Soluble HLA-DR alpha 2 domain inhibits IFN-γ cytokine expression and phosphorylation of STAT4, but not STAT6. A. Human PBL were activated with PHA for 48 h and co-cultured with either sHLA-DRα2 or control protein. Supernatants were subjected to quantitative sandwich ELISA. sHLA-DRα2 at a concentration of 50 µg/ml significantly inhibited the IFN-γ expression of stimulated PBL, whereas control protein exhibited no significant effect. B. No inhibition of IL-10 expression was observed. The results shown represent the means of five independent experiments, respectively. C. Human PBL were allo-activated for 4 h in the presence of anti-TIRC7 mAb and subjected to Western blot analysis. Equal volume of cells were fractionated by SDS-PAGE, and the phosphorylation status of STAT4 and STAT6 was determined by Western blot analysis. STAT4 phosphorylation was decreased in the presence of sHLA-DRα2 whereas pSTAT6 remained unchanged. No changes were observed using STAT4, and anti-Tubulin mAb. Shown is one representative experiment out of three independent experiments. D. Human PBL were isolated using standard Ficoll gradient centrifugation protocol. Cells were activated with PHA, anti-CD3/CD28 mAb, and MLR, respectively, and co-cultured with either sHLA-DRα2 or control protein in varying concentrations (50, 100, and 150 µg/ml). Cells were subjected to CFSE proliferation assays. A significant inhibition of proliferation was observed using sHLA-DRα2 in all proliferation assays whereas the control protein did not show any inhibition. The results shown represent the means of four independent experiments, respectively. E. The inhibition of proliferation of 48 h anti-CD3/CD28 stimulated human PBL by sHLA-DRα2 (100 µg/ml) was prevented by co-incubation of the anti-TIRC7 mAb 136 (100 µg/ml). F. Cell lysates were prepared from 1 h allo-activated PBL and immunoprecipitated (IP) with anti-TIRC7 mAb and subjected to western blot analysis. The immunoblot (IB) with anti-TIRC7 and anti-SHP-1 mAb showed the co-precipitation of SHP-1 and TIRC7. G. The phosphorylation of TCR-ζ chain and ZAP70 induced by IL-2 is inhibited by sHLA-DRα2. Human PBL were isolated and activated using anti-CD3/CD28 mAb for 18 h in the presence and absence of sHLA-DRα2. In the presence of sHLA-DRα2 (50 µg/ml) the phosphorylation of TCR-ζ chain and ZAP70 in stimulated cells (right lane) was reduced to a level similar to that of non-stimulated cells (left lane) while in stimulated cells without sHLA-DRα2 (middle lane) substantial phosphorylation of both proteins was observed.

To examine whether interaction of HLA-DR alpha 2 and TIRC7 results in modulation of cellular responses upon cell activation we studied the effect of sHLA-DRα2 on cytokine expression in PBL activated for 48h with PHA. Incubation of lymphocytes with sHLA-DRα2 resulted in significant inhibition of IFN-γ (Figure 2A). In contrast, IL-10 expression was not affected (Figure 2B). The IFN-γ response is regulated by transcription factors of the STAT (signal transducers and activators of transcription) family [4]. Specifically, STAT4 regulates IFN-γ responses whereas STAT6 has been demonstrated to be involved in the regulation of IL-10 or IL-4 expression [5]. To examine whether the inhibitory response induced by sHLA-DRα2 crosslinking to TIRC7 involves STAT4 and STAT6, human PBL were stimulated with allo-antigen, cultured with sHLA-DRα2 and lysates were analyzed for phosphorylation of STAT4 and STAT6 proteins by Western blot analysis. Indeed, STAT4 phosphorylation was decreased in the presence of sHLA-DRα2 whereas pSTAT6 remained unchanged after 4 h of allostimulation of human lymphocytes (Figure 2C).

### Interaction of sHLA-DRα2 and TIRC7 inhibits proliferation and results in decreased phosphorylation of TCR associated signaling molecules

To further examine cellular responses induced by the interaction of HLA-DR alpha 2 and TIRC7, we studied the effect of sHLA-DRα2 on proliferation of human lymphocytes stimulated by PHA, anti-CD3/CD28, and alloactivation (mixed lymphocyte culture, MLR), respectively. As determined by BrdU incorporation, incubation of lymphocytes with sHLA-DRα2 resulted in a concentration-dependent inhibition of proliferation in these proliferation assays (Figure 2D). To demonstrate the specificity of HLA-DRα2 binding to TIRC7 a displacement ELISA was performed using an anti-TIRC7 monoclonal antibody (mAb136) which was generated by immunization of mice with TIRC7 protein. This antibody binds specifically to TIRC7 and prevents binding between TIRC7 and HLA-DR as observed in Elisa assays (data not shown). The antiproliferative signals induced by binding of sHLA-DRα2 to TIRC7 in human lymphocytes were prevented by blocking the binding of sHLA-DRα2 to TIRC7 using mAb 136 (Figure 2E). The inhibitory effects induced by TIRC7 ligation could be mediated by the recruitment of the phosphotyrosine phosphatase SHP-1 which plays an important negative regulator role on lymphocyte signaling pathways for a variety of receptors involved in lymphocyte activation [6]–[9]. SHP-1 was shown to bind via the ITIM (immunoreceptor tyrosine-based inhibitory motif) motif to cell surface molecules with signaling capacity resulting in the down-regulation or termination of signal transduction events [8], [9]. The hypothesis that SHP-1 might be involved in TIRC7 signaling was based on the fact that an ITIM motif (VIYKWL, aa 371-377) was identified within the TIRC7 protein which would potentially allow for the recruitment of SHP-1. Using an anti-TIRC7 mAb, immune precipitation of lysates from allo-activated PBL revealed co-precipitation of TIRC7 and SHP-1 (Figure 2F). Thus, the recruitment of SHP-1 by TIRC7 upon cell activation is a possible mechanism for the down-regulation of signaling events. SHP-1 was previously demonstrated to induce the dephosphorylation of ZAP70 causing an inhibition of proximal TCR-induced signaling events [10], [11]. To examine whether the interaction of HLA-DR alpha 2 and TIRC7 affects the phosphorylation of ZAP70 and the TCR-ζ chain, freshly isolated human PBL were alloactivated in the presence or absence of sHLA-DR α2 and subjected to immunoblot analysis. Indeed, the phosphorylation of TCR-ζ chain and ZAP70 were found to be decreased to a level similar to that of non-activated cells, whereas the expected phosphorylation was observed in activated lymphocytes not treated with sHLA-DRα2 (Figure 2G). These results indicate that the mechanisms which lead to an inhibition of cell proliferation by the interaction of HLA-DR alpha 2 and TIRC7 involve proximal TCR-induced signaling events.

### Interaction of sHLA-DRα2 with TIRC7 expressed in CD4+ and CD8+ T cells results in cell cycle arrest and apoptosis in lymphocytes

**Figure 3:**
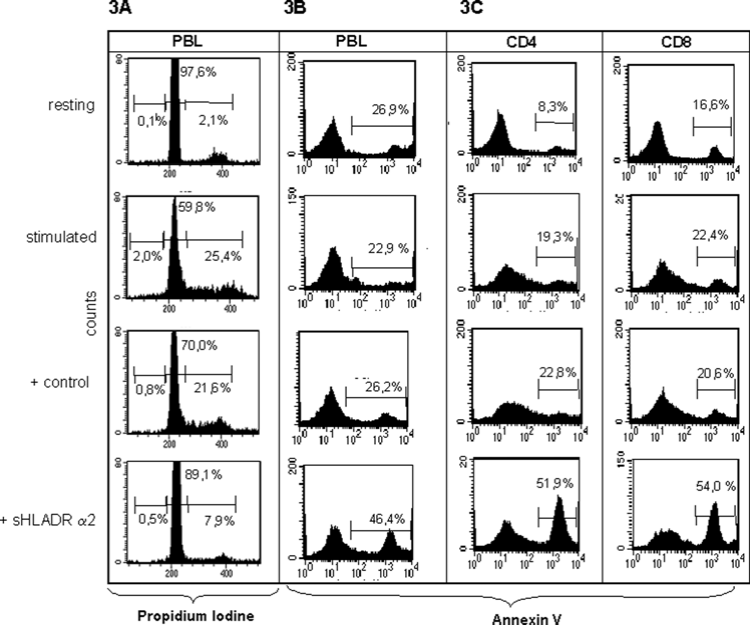
TIRC7 targeting leads to cell cycle arrest and apoptosis. A. Human PBL were activated with anti-CD3/CD28 antibodies for 48 h in the presence of sHLA-DRα2 or control protein (50 µg/ml). The cells were then fixed with 70% ethanol and stained with propidium iodine for cell cycle analysis using flow cytometry. Incubation with sHLA-DR α2 resulted in a G0/G1 arrest (live cell gate) in comparison to control protein. Shown are resting cells, PBL activated with anti-CD3/CD28 for 48 h, activated PBL incubated with control protein, and activated PBL incubated with sHLA-DRα2. B. Human PBL activated with anti-CD3/CD28 were incubated with sHLA-DRα2 or control protein (50 µg/ml) for 48 h and then stained with Annexin V for the analysis of apoptosis using flow cytometry. When incubated with sHLA-DR α2, human PBL showed a strong increase of Annexin V positive cells in comparison to control protein. Apoptosis in stimulated cells prior to the administration of either control protein or sHLA-DRα2 protein was similar in both reactions indicating that the effect on cell cycle distribution and apoptosis was specific. C. Human CD4 and CD8 positive lymphocytes were separated by magnetic beads and activated with anti-CD3/CD28 antibody for 72 h in the presence of either 50 µg/ml sHLA-DRα2 or Fc control protein. The cells were subjected to flow cytometry analysis and stained with Annexin V for the detection of apoptosis. Both, CD4+ and CD8+ cell populations exhibited substantial apoptosis. Shown is one representative experiment out of four independent experiments.

In addition to the induction of anergy and inhibition of proliferative response, cell cycle arrest as well as apoptosis are further important mechanisms to control the activation of lymphocytes [12]. To examine whether the antiproliferative effect induced by sHLA-DRα2 crosslinking to TIRC7 involve these mechanisms as well human PBL were stimulated with anti-CD3/CD28 antibodies in the presence of sHLA-DRα2 or control protein, and analyzed for cell cycle arrest and apoptosis using flow cytometric analysis. Flow cytometry profiles of PBL illustrate that, as a result of stimulation, 25% of the lymphocytes proceeded into the G2/S phase (Figure 3A). In contrast, targeting of TIRC7 with sHLA-DR α2 led to a dramatic reduction of the cell number in G2/S phase as most of the cells prevailed in G0/G1 cell cycle phase (89%) (Figure 3A) which is indicative for cell cycle arrest. Moreover, 72 h after incubation of the cells with sHLA-DRα2 an increased number of cells (46,4%) belonged to the subdiploid population, indicating that cells were apoptotic and died as a consequence of TIRC7 engagement (Figure 3B).

It is well known that HLA-DR molecules interact with CD4 + T cells. Yet from the above results we concluded that HLA-DR protein, especially the alpha 2 domain may induce apoptosis also in CD8+ cells. To examine this hypothesis CD4+ and CD8+ cells were separated by magnetic beads and stimulated 72 h by anti-CD3/CD28 antibodies in the presence and absence of sHLA-DR α2. The results illustrate that compared with non-treated controls sHLA-DRα2 binding leads to induction of apoptosis in both, CD4+ (51%) and CD8+ (54%) cells (Figure 3C).

**Figure 4:**
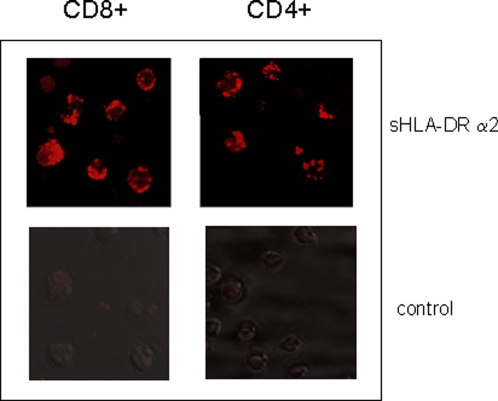
sHLA-DR α2 binds to TIRC7 expressed in CD4 and CD8 T cells. CD4+ and CD8+ T cells were separated by magnetic beads, stimulated with anti-CD3/CD28 mAb for 48 h, and incubated with either sHLA-DR α2 or control protein for 30 min prior to confocal microscopic analysis. Using anti-human Fc-Cy3 mAb as secondary antibody, binding of sHLA-DRα2 or control protein to TIRC7 protein was analyzed. The results show a binding in CD4+ and CD8+ human T cells co-incubated with sHLA-DRα2 (upper panel) whereas no binding was observed in various control experiments using either control protein (lower panel) or anti-Fc-Cy3 conjugated secondary mAb only. Shown is one experiment out of three.

Since interaction of HLA-DR especially with CD8+ T cells has not been described so far, we analyzed the HLA-DRα2 binding to CD8+ and CD4+ T cells. Confocal microscopy illustrates that sHLA-DRα2 exhibits a similar binding pattern on CD4+ (30–40%) and CD8+ (40–50%) cells (Figure 4) as was observed with anti-TIRC7 antibody [1]. No binding was observed in any control experiments, thus, sHLA-DRα2 binds specifically to CD4+ and CD8+ T cells leading to apoptosis in both subsets.

### Targeting of TIRC7 leads to apoptosis via the activation of the intrinsic apoptotic pathway in lymphocytes

**Figure 5:**
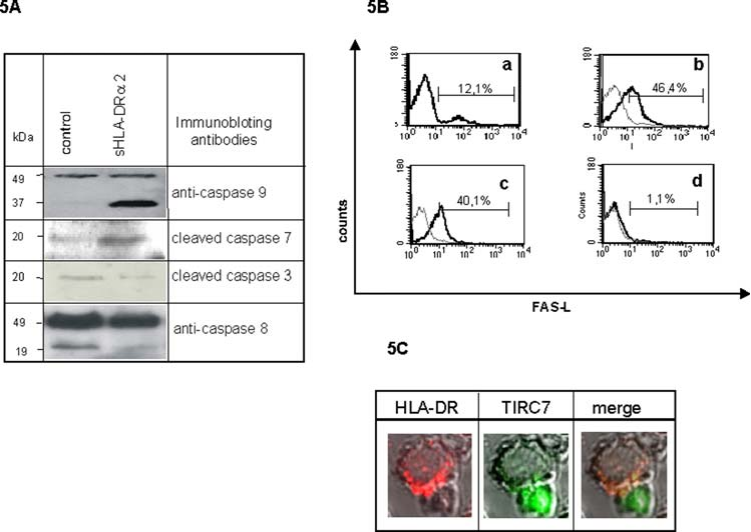
Targeting of TIRC7 in lymphocytes induces apoptosis via the mitochondrial intrinsic pathway. A. PBL were co-incubated 50 µg/ml sHLA-DRα2 or control protein for 5 h, and immunobloting using mAb against caspase 9, caspase 8, caspase 7 and caspase 3 was performed. Activation of caspase 9 and caspase 7 is indicated by the appearance of cleaved fragments with a size of 37 and 20 kDa, respectively. No activation of caspase 8 and 3 was observed. B. Human PBL were isolated, activated with anti-CD3/CD28 antibodies (a) cultured in the presence of 50 µg/ml sHLA-DRα2 (d), and subjected to flow cytometric analysis. In comparison to non-treated controls (b,c) stimulated human T cells incubated with 50 µg/ml sHLA-DRα2 (d) demonstrated a remarkably reduced expression of FasL as detected by FITC labeled anti-FasL mAb. C. Human PBL were recall antigen stimulated for 6 days and incubated with FITC labeled anti-TIRC7 mAb. A monocyte cell line, THP-1, was incubated with anti-HLA-DR mAb and secondary stained with Cy3-conjugate. To increase cell interaction, PBL and THP-1 cells were mixed and incubated at 37°C for 20 min and subjected to confocal microscopic analysis. The data show that HLA-DR (red color) and TIRC7 (green color) co-localize (merge, yellow color) at the physical site of T cell/APC interaction. Shown is one experiment out of three.

Proteins central to apoptosis are members of the caspase family which can be divided into initiator (caspase 8 and 9) and effector (caspase 7 and 3) caspases [13]. In the intrinsic pathway the apoptosis is mediated by mitochondria whereas the extracellular death receptor, FasL, mediates the extrinsic pathway [13], [14]. To analyze the mechanistic details, human PBL was used to test the caspase activity after 5 h of TIRC7 targeting using sHLA-DRα2. Caspase 9 was activated as was indicated by an increase of the cleaved product in the presence of sHLA-DRα2 whereas no activation of caspase 8 was observed in PBL (Figure 5A). Analysis of caspase 7 showed an increase of the cleaved product of 20kDa size upon TIRC7 ligation, while an activation of caspase 3 was not observed (Figure 5A). To analyze whether TIRC7-dependent apoptosis can be reversed by adding inhibitor of caspase 9 to the cultures, incubation of cells with the caspase 9 inhibitor z-LEH-fmk reduced TIRC7-dependent apoptosis to normal levels (data not shown). Although no activation of caspase 8 was observed after sHLA-DRα2 ligation, modulation of the FasL expression on T cells was analyzed. Flow cytometry analysis of PBL activated with anti-CD3/CD28 antibody for 24 h in the presence of the sHLA-DRα2 revealed a remarkably reduced expression of FasL at the cell surface of human T cells (Figure 5B, d).

These results are in agreement with a down-regulatory effect of TIRC7 on ZAP70 activity, since ZAP70 was shown to be essential for the regulation of FasL expression on the surface of activated T cells [11] and indicate that TIRC7 mediated modulation of the proliferative response in PBL involves the intrinsic, mitochondrial pathway via caspase 9.

### HLA-DR co-localizes with TIRC7 at the APC-T cell interface and soluble HLA-DRα2 prevents APC mediated T cell cytokine release in vitro and in vivo

Upon activation of T lymphocytes both, HLA proteins [15] and TIRC7 [16] cluster at the site of T cell/APC junction. To examine the distribution of both molecules at the site of cell – cell interaction, human PBL were activated with recall antigens for 72 h. Confocal microscopy showed that in recall antigen activated human lymphocytes both, TIRC7 (green) and HLA-DR (red), concentrated at the site of the cell – cell interaction. An overlay of both immunostains suggested a co-localization of both molecules (yellow) (Figure 5C).

**Figure 6:**
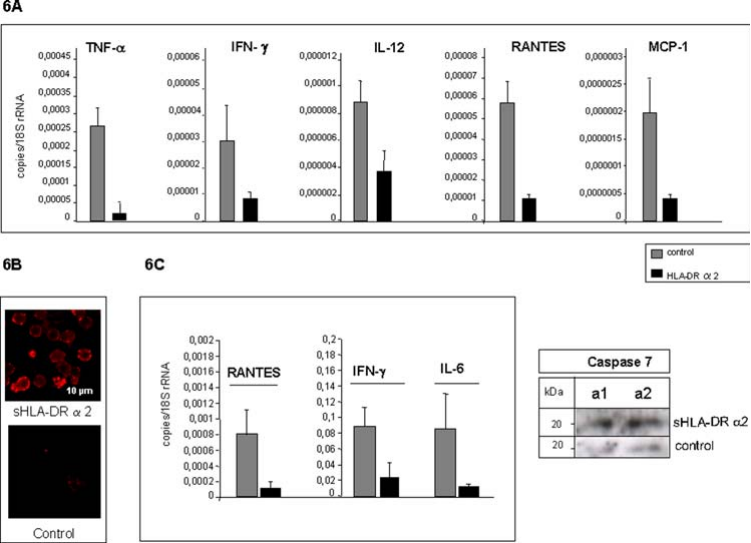
TIRC7 signals triggering prevents T cell - APC interaction reflected in inhibition of several cytokines. A. Human PBL were isolated using standard Ficoll gradient centrifugation protocol and culture 14 days to induce monocyte differentiation. Either sHLA-DRα2 (50 µg/ml) or control (50 µg/ml) were co-incubated and subjected to cytokine release assays. Cytokine levels were measured by quantitative real time PCR which revealed a profound inhibition of MCP-1, IL-12, IFN-γ, TNF-α and Rantes expression compared with the control. B. Human sHLA-DRα2 showed specific cross-reactivity on balb/c mice splenocytes (left side). For the in vivo functional analysis balb/c splenocytes were isolated and further subjected to microscopic analysis for cross-reactivity of human sHLA-DRα2 to mouse TIRC7 which resulted in significant binding on activated splenocytes. C. In balb/c mice (n = 14) LPS was administered followed by a single dose (100 µg/mice, i.p.) of human sHLA-DRα2 (200 µg/day, i.p.). Human sHLA-DRα2 resulted in a significant down-regulation of IFN-γ, TNF-α and Rantes after 24 h treatment. sHLA-DRα2 or control protein treated splenocytes were subjected to immunobloting using mAb against caspase 7. Activation of caspase 7 is indicated by the appearance of cleaved fragments with a size of 20 kDa in sHLA-DRα2 treated animals (a1 and a2) whereas no activation of caspase 7 was observed in controls. Shown are two examples out of five.

Functional importance of T cell activation through crosslinking with MHC class II molecules bearing APC is the induction of IL-12 expression which leads to an increased IFN-γ expression in T cells [4]. IFN-γ acts itself as an activator of APC which in turn induces IL-12 subsequently resulting in inflammation. Data obtained from our studies indicates that TIRC7 and HLA DR alpha 2 interaction controls APC - T cell interaction by induction of negative regulatory signals and prevents an excess of APC - T cell interaction during immune activation. We therefore hypothesize that triggering TIRC7 signals via sHLA-DRα2 should suppress the APC - T cell interaction and prevent associated cytokine release upon stimulation. To prove this hypothesis in vitro, we used the model of LPS induced APC activation. Macrophages exposed 48 h to either sHLA-DRα2 or control protein in the presence of LPS and subsequently subjected to analysis of cytokine expression via real time PCR method. The results show a profound reduction of cytokines such as IFN-γ, TNF-alpha, IL-12. Interestingly, chemokines such as MCP-1 and Rantes were also inhibited indicating that TIRC7-HLA-DR pathway is controlling T cell and APC specific cytokines after immune activation (Figure 6A).

Lipopolysaccaride application in mice is characterized by rapid activation of APC which induces IL-12, endoxinemia and enhanced cytokine induction such as IFN-γ. Of particular relevance are previous studies in which in vivo neutralization of IFN-γ prevented lethal hypersensitivity reactions to LPS and reduced IFN-γ-dependent lethality [17]. Also, lymphocytes from mice deficient for IFN-γ have been shown to release markedly reduced amounts of proinflammatory cytokines [18].

Due to immediate activation of APC and T cells, we utilized the LPS induction model in mice to prove the physiological relevance of the sHLA-DRα2 targeting of TIRC7 in vivo. First, cross-reactivity studies were performed using mouse splenocytes and demonstrated that human sHLA-DRα2 specifically binds to TIRC7 in mice as shown in microscopic analysis whereas control Fc did not exhibit any binding (Figure 6B, left panel). Immediately after the LPS application in vivo, mice were treated with either sHLA-DRα2 or control protein and splenocytes were subjected to cytokine analysis. In this study, as assessed by PCR analysis, splenocytes of mice treated with a single dose of sHLA-DRα2 at the time of intravenous immune challenge with lipopolysaccaride showed substantially reduced IFN-γ, TNF-α, Rantes and IL-6 production 24 h after activation compared with control mice (Fig 6B, right panel). Thus, treatment with sHLA-DRα2 resulted in inhibition of inflammation induced via activation of APC and T cells. Splenocytes of mice were also subjected to western blots for the analysis of caspase activation. As demonstrated in Figure 6C, splenocytes exposed to sHLA-DRα2 treatment showed an increased activation of caspase 7 whereas controls do not exhibit any activation of caspase 7, indicating that treatment with sHLA-DRα2 is able to mediate apoptosis in vivo.

## Discussion

The beta-2 domain of the conserved HLA-DR alpha molecule encoded by the HLA-DRA1 locus interacts with the CD4 molecule in human lymphocytes [19]. The T-cell receptor binds to polymorphic beta 1 domains of HLA-DR with associated peptide antigen [20]. Other molecules which bind to HLA-DR include super antigens such as staphylococcus enterotoxin B (SEB) [21]–[23], and toxic shock syndrome toxin (TSST-1) [24]–[26]. These molecules bind to the alpha 1 and beta 1 domains of HLA-DR molecules, respectively [26]. The ability of SEB to bind many different DR alleles can be explained by its interaction with the DR alpha 1 chain, which is conserved in all DR molecules [23]. TSST-1 extends over nearly half of the binding groove and contacts the alpha helix of the alpha 1 domain of DR protein [25]–[27]. The HLA-DR alpha 2 domain, which is highly conserved in humans, has not been heretofore recognized as a ligand for other molecules.

Several conclusions can be drawn from our work. The first novel aspect of the work presented here is the identification of the interaction between HLA-DRα2 and TIRC7, and the demonstration of functional relevance of this binding. Soluble HLA-DRα2 delivers distinct and selective signals via inhibition of IFN-γ, but not IL10 response, strong inhibition of proliferation and induction of apoptosis. The inhibitory effect observed in the lymphocyte cultures is solely due to binding between HLA-DR alpha 2 and TIRC7 as the specific anti-TIRC7 mAb prevents the inhibition of immune activation.

The second novel aspect of our work relates to the finding that the HLA-DR protein, beyond its well-known role in initiating lymphocyte activation by presenting peptides to the TCR (Figure 4F, left panel) is also able to induce negative signals via binding of its non-polymorphic alpha 2 domain to TIRC7 (Figure 4F, left panel). This binding is not restricted to CD4+ cells but also includes CD8+ cells and B cells. The findings of dampening effects on cell activation provided by the HLA-DR protein and its binding to cell surface molecules other than CD4 and TCR substantially extends the understanding of the regulation of the immune activation process and the interactions involved. It is unclear whether the co-localization of both molecules at the site of cell interaction, as is shown in the present manuscript, may indicate also cis-acting ligation of TIRC7 and HLA-DR on the same cell and whether such interaction bears functional relevance. Our data, however, demonstrate unanimously that the trans-acting ligation between both molecules induces strong antiproliferative effects in various lymphocyte populations.

The third aspect of this work relates to the finding that the strong antiproliferative response triggered by TIRC7 ligation is induced on at least two levels of interference. On the one hand, the antiproliferative effect is induced by dephosphorylation of early signaling molecules such as TCR-ζ and ZAP70 which is associated with down-regulation of FasL. This result is in accordance with the fast upregulation of TIRC7 at the cell membrane upon lymphocyte activation and the clustering of the molecule at the cell – cell adhesion site [16] where the immunological synapse is formed [15]. Thus, TIRC7 mediates antiproliferative signals early in the activation process. On the other hand, the induction of apoptosis via caspase 9 indicates that the pro-apoptotic pathways include additional important mechanisms which are orchestrated, at least in part, by the TIRC7 engagement with HLA-DR alpha 2. In accordance with this FasL which is the extracellular receptor of the extrinsic apoptotic pathway via caspase 8 was shown to be downregulated. After stimulation, in splenocytes obtained from TIRC7 deficient mice apoptosis was decreased in comparison to WT splenocytes (unpublished observation) indicating that TIRC7 is central to the induction of apoptosis in lymphocytes. These results also support the agonistic effect of the compounds used in this study on the TIRC7-HLA-DRα2 pathway.

The fourth aspect of our work relates to the observation that binding between HLA-DRα2 and TIRC7 is functional in T cells and APC. Crosslinking of TIRC7 with HLA-DRα2 in T cells and macrophages demonstrates that TIRC7-HLA-DR interaction mediated signals control the expression of proinflammatory cytokines such as IL-12 which naturally potentiates inflammation via the induction of IFN-γ expression. The data obtained from the LPS induction in vivo demonstrate that the anti-inflammatory and apoptotic mode of action of sHLA-DRα2 is physiologically relevant. The reduction of cytokines such as IL-6, Rantes and IFN-γ in cells obtained from mice treated with HLA-DRα2 indicates that TIRC7 targeting might be potentially translated into clinical use to prevent acute inflammatory response.

The clinical data and cytokine expression results obtained from acute inflammatory disease, LPS induction, in mice demonstrate that treatment with sHLA-DRα2 can control inflammatory conditions supporting the anti-inflammatory mode of action of the protein. Accordingly, a substitution of the signal to modulate TIRC7 pathway using sHLA-DRα2 might lead to a therapeutic approach unifying both, T cell and APC therapeutic targeting as TIRC7 is expressed in 30% of all lymphocytes.

**Figure 7:**
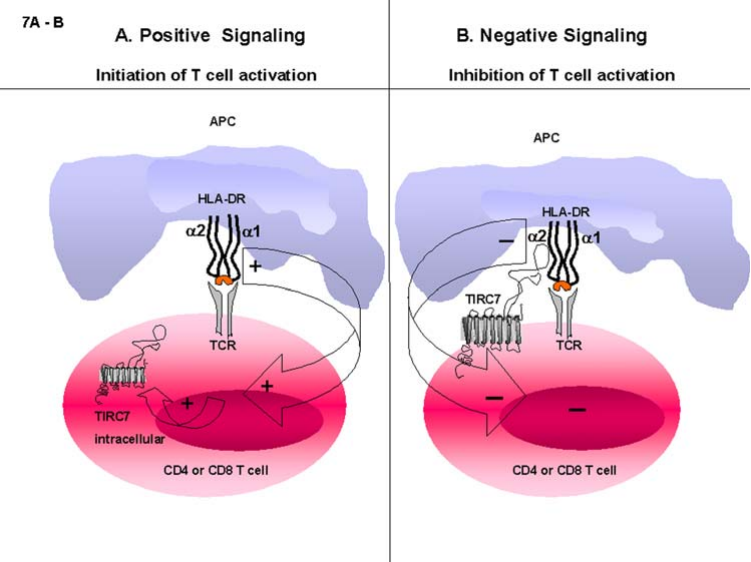
Scheme of proposed model of regulation of immune activation via TIRC7-HLA-DR alpha 2 binding. TIRC7 serves as ligand for HLA-DR alpha 2 upon TCR activation in the early phase of immune activation. After positive signals were received and immune cells are activated, TIRC7 is expressed on the cell surface (A) and its binding to HLA-DR alpha 2 transduces negative signals to lymphocytes (B).

In summary, this work provides novel data for the interaction between HLA-DR alpha 2 and TIRC7 and the functional relevance of this binding in lymphocytes in vitro and in vivo after immune activation. For the first time, it is here reported that the HLA-DR molecule, which is classically described to initiate the cellular immune response also mediates inhibitory signals and apoptosis via binding to TIRC7 in lymphocytes, thereby modulating the decisive first phase of the immune response (Figure 7). This work introduces HLA-DR as a molecule with a dual regulatory function in lymphocytes which might have the potential for the development of novel therapeutic approaches to treat immune mediated diseases.

## Methods

### Yeast two-hybrid screen

For bait construction, DNA fragments of TIRC7 containing the N-terminus (aa 1-173), large extracellular domain (aa 438-512) and C-terminus (aa 586-614) were amplified by PCR and cloned into the pBD-GAL4 Cam vector, thereby generating an in-frame fusion with the GAL4-DNA binding domain. A human PBL cDNA library was constructed using HybriZAP 2.1 Two-Hybrid cDNA Library Kit (Stratagene). Standard yeast techniques were used to manipulate strains. To confirm the observed interaction the obtained plasmids were tested in MATCHMAKER GAL4-Two-Hybrid System3 (Clontech).

### Immunoprecipitation, Western blot

Lysates from allo-activated PBL and Jurkat cells were incubated with anti-TIRC7 mAb (20 µg) and mouse IgG as control (Calbiochem) followed by Western blot analysis using anti-HLA-DR mAb (CBL120, Cymbus Biotechnology) or anti-TIRC7 mAb. To analyze phosphorylation of STAT proteins, alloactivated PBL were incubated with 50 µg sHLA-DR α2 for 4 h. Lysates were subjected to Western blot analysis using mAb against either anti-phospho-STAT4 (Ser 721) or (p-STAT4 Santa Cruz, dilution 1/1000 in 5% BSA/PBS) or STAT4 or STAT6 (Santa Cruz, dilution 1/1000 in 5% BSA/PBS). To analyze phosphorylation of TCR-ζ (Santa Cruz) and ZAP70 (Cell Signaling) PBL were stimulated with with 100 U/ml IL-2 for 18 h. Western blots were performed by incubation with a mouse anti-human p-TCR-ζ antibody or p-ZAP70. An anti-mouse POD antibody was used for final analysis in an ECL detection system. For immunoprecipitation studies with SHP1, lysates were incubated for 6h at 4°C with anti-TIRC7 mAb (20 µg), in the presence of followed by incubation with protein-A/protein-G Sepharose beads (Calbiochem) overnight, at 4°C. Immunoprecipitates were analyzed by immunoblotting with anti-TIRC7 mAb or anti-SHP1 diluted of 1∶200 in 5% milk/PBS and were subjected to chemiluminescent detection (Amersham Pharmacia). For caspase assays PBL were seeded at a density of 1,5×10E7 cells. sHLA-DR α2 or control protein were added at a concentration of 50 µg/ml. Cells were incubated for 6 h, harvested, washed and frozen in liquid nitrogen. Cell lysis was performed with 50 mM Pipes-HCl, pH 6,5, 2 mM EDTA, 0,1% CHAPS, 10 mM NaF, 5 mM DTT and protease inhibitors. Supernatants were boiled with Laemmli-buffer and subjected to SDS-PAGE. Gels were blotted onto PVDF membranes and analysed using specific antibodies (anti-caspase 8, 9, 3 and 7) (Cell Signaling).

### Proliferation and apoptosis assay

For proliferation assay, PBL were obtained from healthy volunteers after written and informed consent had been obtained. PBL were isolated according to the Ficoll-Paque density centrifugation protocol. PBL were labeled by incubation with CFSE (Carboxyfluorescein succinimidyl ester). The CFSE-labeled PBL were stimulated with PHA (1 µg/ml, Sigma) or anti-CD3/CD28 mAb (10 µg/ml) and incubated for 3 days at 5% CO_2_, at 37°C in the presence of sHLA-DR α2 or control protein, respectively, at a concentration ranging between 50–150 µg/ml. For alloactivation, mixed lymphocytes culture reaction was performed using donor cells which were inactivated and incubated with equal cell number of recipient lymphocytes (200.000 total cells/well) for 72 h at 5% CO_2_, at 37°C. These cultures were incubated with either with sHLA-DR α2 or control protein at a concentration of 50 and 100 µg/ml. The proliferation rate of PBL was determined by FACS analysis. For proliferation assays, various human T- and B- cell lines were incubated for 48 h at 37°C, 5% CO_2,_ in the presence of chimeric anti-TIRC7 mAb or control mAb (50 µg/ml), labelled with 10 µl/well BrdU labelling solution and incubated for 16 h at 37°C. Cell Proliferation ELISA BrdU-Kit (Roche Diagnostics GmbH) was used. The measurement of the samples was performed 10–30 minutes after substrate addition at 370 nm (reference wavelength: 492 nm) in an ELISA reader. For detection of apoptosis detection, human PBL or cell lines were stained with 7-AAD for 20 minutes at RT. Samples were measured and analyzed by flow cytometry.

### ELISA cytokine analysis

PBL of human healthy donors isolated according to the Ficoll-Paque density centrifugation protocol were incubated with 1 µg/ml PHA (Sigma) in 5% CO_2_, at 37°C for 48 h in the presence of sHLA-DRα2 and control protein, respectively. IFN-γ or IL-10 was quantified in supernatants of PHA stimulated cells. Samples were run in triplicates on 96-well microtiter plates. Cytokine level was determined using the Cytoscreen® ELISA Kit (Biosource).

### Flow cytometry and confocal microscopy

Splenocytes from wild type (WT) and TIRC7(-/-) mice were isolated with a cell strainer and transferred to 15 ml tubes. Cells were stimulated with 4 µg/ml ConA (Sigma) for 14–16 h and permeabilized with Perm-solution2 (BD biosciences) and Fc-blocked for 30 min at 4°C. After incubation with 8 µg/ml HLA-DR Fc or control protein for 30 min, cells were secondary stained with anti-human Cy3 (Sigma) (1∶250) and analyzed via FACS Calibur (BD Biosciences). Isolated human PBL (5×10^5^ cells/well) were incubated with 50 µg/ml soluble HLA-DR alpha 2 or control protein. After 72 h or 5 h of incubation the cells were washed with FACS-buffer and stained with 2,5 µl FAS-L-PE or caspase 7 (BD Biosciences) or mIgG-PE as control for 30 min at RT. Immunofluorescence analysis were performed using standard protocols. All images were taken using LSM 510 confocal laser microscope (ZEISS).

### Expression of TIRC7-myc fusion protein and sHLA-DR α 2-Fc fusion protein in COS7 cells

TIRC7-*myc* fusion protein [1] was expressed in COS7 cells after transient transfection with a pCDNA3 construct containing an expression cassette for TIRC7-*myc* fusion protein using Fugene6 Transfection Reagent (Roche). For HLA-DR alpha 2 expression in mammalian expression system the HLA-DR alpha 2 domain was fused to IgG1-Fc fragment. COS7 cells were transiently transfected with vector and after 96 h the fusion protein was purified by Sepharose A column from cell culture supernatants.

### Real time PCR

PML were separated from buffy coats of healthy donors by centrifugation on a Ficoll-Paque density gradient and monocytes were purified by adherence. After 14 days, we stimulated monocytes with 5 µg/ml LPS and 200 u/ml IL-4 and incubated them with 50 µg/ml HLA-DR-Fc or control for 2 days. RNA was extracted using RNeasy Mini Kit (Qiagen). cDNA was synthesized from mRNA with random hexamers and TaqMan reverse transcriptase (Applied Biosystems). Reactions use specific primers and Sybr Green PCR Master Mix (Applied Biosystems) or specific probes (TaqMan Gene Expression Assay, Applied Biosystems), detected by use of an ABI Prism 7300 Sequence Detection System (Applied Biosystems). To standardize results, we expressed them as the number of target gene copies per 10^5^ copies of 18S-rRNA.

### LPS and induction in mice

Balb/C mice (10–14 weeks old) were induced intraperitoneally with 50 µg LPS on day 0. Immediately afterwards 14 mice were treated intraperitoneally with either sHLA-DR α2-Fc (200 µg in PBS) or human Fc as control (200 µg in PBS), respectively. After 24 h, spleens were removed for further cytokine analysis via FACS.
